# Conversion of Free Fatty Acid in *Calophyllum inophyllum* Oil to Fatty Acid Ester as Precursor of Bio-Based Epoxy Plasticizer via SnCl_2_–Catalyzed Esterification

**DOI:** 10.3390/polym15010123

**Published:** 2022-12-28

**Authors:** Ratna Dewi Kusumaningtyas, Haniif Prasetiawan, Nanda Dwi Anggraeni, Elva Dianis Novi Anisa, Dhoni Hartanto

**Affiliations:** Chemical Engineering Department, Faculty of Engineering, Universitas Negeri Semarang, Sekaran, Gunungpati, Semarang 50229, Indonesia

**Keywords:** *Calophyllum inophyllum* seed oil, SnCl_2_.2H_2_O, fatty acid ester, response surface methodology, epoxy plasticizer

## Abstract

The preparation and application of bio based plasticizers derived from vegetable oils has gained increasing attention in the polymer industry to date due to the emerging risk shown by the traditional petroleum-based phthalate plasticizer. Epoxy fatty acid ester is among the prospective alternative plasticizers since it is ecofriendly, non-toxic, biodegradable, low migration, and low carbon footprint. Epoxy plasticizer can be synthesized by the epoxidation reaction of fatty acid ester. In this study, the preparation of fatty acid ester as a green precursor of epoxy ester plasticizer was performed via esterification of free fatty acid (FFA) in high acidic *Calophyllum inophyllum* Seed Oil (CSO) using methanol in the presence of SnCl_2_.2H_2_O catalyst. The analysis of the process variables and responses using Box–Behnken Design (BBD) of Response Surface Methodology (RSM) was also accomplished. It was found that the quadratic model is the most appropriate model for the optimization process. The BBD analysis demonstrated that the optimum FFA conversion and residual FFA content were 75.03% and 4.59%, respectively, achieved at the following process condition: a reaction temperature of 59.36 °C, a reaction time of 117.80 min, and a catalyst concentration of 5.61%. The fatty acid ester generated was an intermediate product which can undergo a further epoxidation process to produce epoxy plasticizer in polymeric material production.

## 1. Introduction

Plasticizer is an important additive in polymer, especially in the plastic industry. The IUPAC definition of plasticizer is a substance included in a material such as plastic or elastomer to enhance its flexibility, working ability, and distensibility. This function can be executed by decreasing the second order transition temperature, also known as the glass transition temperature [1]. Plasticizers are low molecular weight molecules sited between the polymer chains that develop a secondary bond with the polymer chains. Thus, they interrupt the hydrogen bond and spread the polymer chains apart, which improves the polymer properties in ways such as lowering the modulus, making the mass character of the material softer, providing better gas permeability, enhancing the degree of crystallinity, and reducing the tension of deformation [2,3]. The demand for plasticizer has notably increased along with the rapid growth of the plastic and polymer industry during the last decade.

To date, the most widely used plasticizers are conventional petroleum-based phthalates, i.e., diisononyl phthalate (DINP), di(2-ethylhexyl) phthalate (DEHP), dibutyl phthalate (DBP), diethyl phthalate (DEP), di-isobutyl phthalate (DIBP), and n-butyl benzyl phthalate (BBP). Phthalates are applied in many polymer products, especially PVC products. However, utilization of phthalate plasticizers has caused problems recently, since they exhibit a negative effect on human health and the environment [4,5,6,7]. Besides, they do not have biodegradable and renewable characteristic. Therefore, it is essential to develop a non-toxic, biodegradable, and renewable plasticizer with good performance which can be used in various polymer products, such as food packaging, consumer goods, electrical insulation, and medical products.

Bio based plasticizers derived from vegetable oils are among the prospective alternative since they have ecofriendly, non-toxic, biodegradable, low migration, and low carbon footprint properties. Various types of bio-plasticizers can be produced from vegetable oil raw materials such as, for instance, epoxidized oil (triglyceride) and epoxidized fatty acid esters [1,6,8]. Among numerous bio based plasticizers, epoxidized fatty acid methyl ester, also known as epoxy fatty acid ester, is favorable for application as an additive material in PVC, which is attributable to its benefits, viz., high plasticizing efficiency, renewability, biodegradability, and cost-effectiveness [9]. Epoxy fatty acid esters have better solubility in the polymeric matrix than epoxidized oil and offer superior elasticity even at low temperatures [10].

Vegetable oil fatty acid esters as precursor of epoxy fatty acid esters can be prepared via two different routes, namely the transesterification of triglyceride and the esterification of free fatty acid. Vegetable oils are mainly composed of triglycerides, which consist of fatty acid units linked to glycerol [11]. Fatty acid esters can be synthesized by transesterification of the triglyceride in the oil using a short chain alcohol such as methanol over an acid or base catalyst [9,12,13]. The nonedible vegetable oils, however, generally contain high free fatty acid (FFA) in addition to the main triglyceride compound. The high FFA content causes the acidic character of the vegetable oil. FFA is usually unfavorable since it has bad odor and makes the oil rancid [14]. The standard quality of commercial vegetable oil such as crude palm oil is required to have an FFA content lower than 5% [15]. In spite of this fact, FFA can be transformed to fatty acid ester via an esterification reaction using short chain alcohols in the presence of an acid catalyst [13,16]. Fatty acid esters synthesized via either triglyceride transesterification or FFA esterification can further undergo an epoxidation reaction to produce epoxy fatty acid esters. Fatty acid esters have a low viscosity; hence they need lower organic solvent in the epoxidation reaction [17].

The epoxidation reaction requires fatty acid ester precursors which comprise a high content of unsaturated fatty esters [10,17]. Epoxidation is a double bond addition reaction, in which the double bonds are transformed into oxirane [7]. Thus, it involves the formation of oxirane (epoxy) through the reaction between the olefinic double bond compound and the peroxyacids or peracids. Epoxides or oxiranes consist of cyclic ethers with a reactive 3-membered ring. Peroxyacids in the epoxidation reaction are generally yielded via the reaction between acetic acid or formic acid with hydrogen peroxide using a strong inorganic acid. It can be also conducted by directly introducing peroxyacid into the reactants mixture. The resulting peroxyacids then convert the double bond into the epoxy. A recent innovation in the area of fatty acid esters conversion to epoxy is enzymatic reaction technology [18,19].

Several works related to the epoxidation of fatty acid esters sourced from various vegetable oils, such as soybean, linseed, rapeseed, castor, grapeseed, avocado, olive, microalgae, RBD palm olein, and sunflower oils [9,17,18,20,21,22] have been extensively reported. However, the synthesis of an epoxy fatty acid ester derived from *Calophyllum inophyllum* Seed Oil has not been broadly studied. *Calophyllum inophyllum* Seed Oil (CSO) is a prospective source of fatty acid esters as precursors of epoxy fatty acid esters. The *Calophyllum inophyllum* plant, locally known as the nyamplung or tamanu tree or beach mahogany, originally comes from Indo-Pacific area (Africa, India, South East Asia, Australia, and Pacific islands) [23]. The *Calophyllum inophyllum* seed is an excellent source of vegetable oil with oil content of 65–75% [24]. Based on our previous investigation, *Calophyllum inophyllum* Seed Oil (CSO) comprises high unsaturated fatty acid. The fatty acids composing CSO are predominantly unsaturated fatty acids (40% oleic acid, 29.94% linoleic acid, and 0.6% arachidic acid) with small portion saturated fatty acid (15.51% palmitic acid and 14.39% stearic acid). CSO is a nonedible oil, containing gum and high FFA content of 19.18% [25]. The undesired high FFA content in CSO has the potential to be converted to fatty acid esters as precursor of epoxy fatty acid ester plasticizer through acid catalyzed esterification using methanol.

In this work, the esterification of the FFA present in CSO with methanol using SnCl_2_.2H_2_O was carried out to produce fatty acid ester as precursor of epoxy fatty acid ester. The heterogeneous SnCl_2_.2H_2_O (tin chloride) catalyst was employed to promote the reaction by reason of its superiority. SnCl_2_.2H_2_O is a low cost Lewis acid catalyst which is tolerant to water, stable, minimally corrosive, and simple to handle. It is milder than Brønsted acid catalyst but capable of providing high catalytic activity. Lewis acids are compounds with lack of electrons which can perform to activate substrate rich in electrons [26,27]. This catalyst also possesses the general advantages of heterogeneous catalyst, specifically easy separation from the product mixture and reusability [28].

To optimize the process condition for the esterification of FFA in CSO with methanol in the presence on SnCl_2_.2H_2_O, a statistical model was applied. Response Surface Methodology (RSM) is a rigorous technique that can be implemented to assess numerous parameters with a minimum number of experiments. It involves a mathematical and statistical procedure to create an experimental design which can examine the influences of the independent process variables on the response variable, thus allowing the optimum response to be verified [29]. In the optimization process, a suitable design should be employed. The models that are applicable for the factorial analysis are Box–Behnken Design (BBD), Doehlert Design (DD) and Central Composite Design (CCD). These models can predict the response function to the actual data using the quadratic function [30]. BBD is more efficient and cost-effective than DD and CCD since it has no extreme points and needs fewer points than the others for the analysis and optimization [31]. The purpose of this work was to determine the proper process condition which results in the highest reaction conversion and the lowest residual FFA by using BBD in RSM for the esterification of FFA in CSO with methanol over SnCl_2_.2H_2_O catalyst. At the optimum process condition, the highest yield of fatty acid esters as precursor of epoxy plasticizer was also achieved.

## 2. Materials and Methods

### 2.1. Materials

*Calophyllum inophyllum* Seed Oil (CSO) was obtained from a local supplier in Central Java, Indonesia. It had an acid value and FFA content of 36.542 mg KOH/g oil and 18.39%, respectively. The most dominant fatty acid composing the CSO was oleic acid, which has a molecular weight of 282.52 g/mol as reported in our previous work [25]. The other materials used were phosphoric acid, methanol (technical grade, purchased from local chemical store), ethanol p.a. (Merck), SnCl_2_.2H_2_O or tin(II)chloride catalyst (Merck), KOH p.a. (Merck), oxalic acid p.a. (Merck), distilled water, and phenolphthalein solution.

### 2.2. Methods

#### 2.2.1. Esterification Reaction

Prior to the esterification reaction, the CSO was degummed using phosphoric acid to remove the phospholipids and mucilaginous gums content [32]. The acid degumming process was performed using a similar method to the previous work [25]. The degummed CSO was then underwent the esterification reaction. Initially, the CSO and methanol were weighed to obtain a molar ratio of CSO and methanol of 1:30. The CSO was heated until it reached the desired temperature (40 °C, 50 °C, and 60 °C) in a three necks flask reactor. At the same time, a certain amount of SnCl_2_.2H_2_O was solved and mixed with methanol in another flask. The SnCl_2_.2H_2_O catalyst employed for the reaction was varied at 1%, 3%, 5%, and 7% *w/w* of CSO. The mixture of methanol and SnCl_2_.2H_2_O catalyst was separately heated up to the similar temperature. Once the targeted temperature was attained, the methanol-SnCl_2_.2H_2_O catalyst mixture was introduced into the reactor, and this was recorded as the initial time of the esterification reaction. The esterification reaction was conducted for 120 min using a batch reactor which was equipped with a condenser and magnetic stirrer. The high agitation speed of 1000 rpm was applied to enhance the mixing of the solid catalyzed reaction [33,34,35]. Samples were taken periodically every 10 min. The samples were tested to determine the acid value using standard carboxylic-acid-titration techniques [36,37]. According to Kurniati et al. [38], The FFA conversion (*X_A_*) at a certain sampling time was determined based on the residual acid value at reaction time t as shown in Equation (1).

(1) $$X_{A}=\frac{AV_{i}-AV_{t}}{AV_{i}}\times 100\%$$

where *X_A_* is the reaction conversion (%), *AV_i_* is the initial acid value (*t* = 0) (mg), and *AV_t_* is the residual acid value at reaction time (mg).

The FFA content was calculated using Equation (2) [39].

(2) $$FFA\;Content\;\left(\%\right)=\frac{A\times N\times MW}{G\times 1000}\times 100$$

where *FFA* Content is the reaction conversion (%), *A* is the volume of KOH (ml), *N* is the normality of KOH (N), *MW* is the average molecular weight of the fatty acids (g/mol), and *G* is the sample weight (g).

#### 2.2.2. Optimization Using Box–Behnken Design of Response Surface Methodology

The experimental data were used for the optimization of the operation condition to obtain the lowest FFA content in the CSO and the highest reaction conversion using Box–Behnken Design (BBD) of Response Surface Methodology (RSM). The simulation was conducted using Design Expert version 13 software. BBD was chosen since it can optimize the parameters effectively with the minimum number of experiments and allows analysis of the interactions between the parameters. In this study, BBD was performed using a total of 15 experimental runs, and the center point measurements were repeated three times to accomplish an accurate calculation of the experimental error. The parameters studied as the independent variables in this work were temperature (A), reaction time (B), and catalyst concentration (C). Each parameter was examined at 3 levels, viz., −1 indicated the low level, +1 represented the high level, and 0 was used as the central point to evaluate the experimental error [40]. The independent variables and their levels are presented in Table 1. Furthermore, the design of the randomized response model is shown in Table 2.

The average magnitude of error between the predicted value and actual value (experimental data) was calculated using Equation (3), in which *MAPE* is Mean Absolute Percentage Error and *n* is the number of data points.

(3) $$MAPE=\sum^{\;}\frac{\left|\frac{predicted\;value-experimental\;data}{experimental\;data}\right|}{n}\times 100\%$$

## 3. Results and Discussion

### 3.1. Effects of the Experimental Variables on the Reaction Conversion

**Figure 1:**
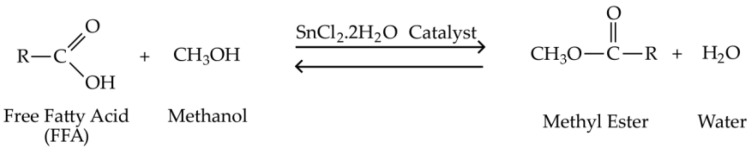
Esterification of FFA with Methanol in the Presence of SnCl_2_.2H_2_O Catalyst.

The esterification of high acidic *Calophyllum inophyllum* seed oil (CSO) with methanol in the presence of SnCl_2_.2H_2_O catalyst to transform free fatty acid (FFA) to fatty acid ester as precursor of bio-based epoxy plasticizer has been conducted in this work. The esterification reaction of FFA in CSO with methanol over SnCl_2_.2H_2_O is illustrated in Figure 1.

**Figure 2:**
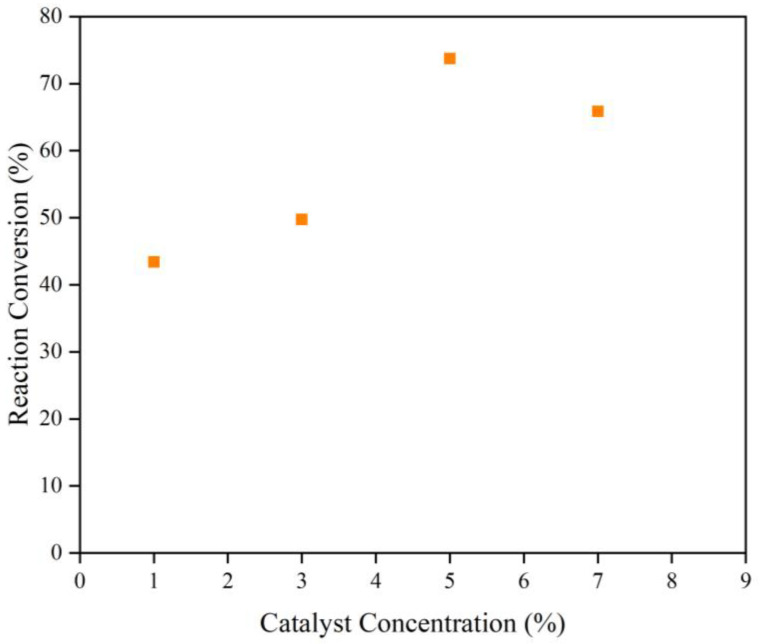
Effect of the Catalyst Concentration on the Reaction Conversion of FFA Esterification in CSO over SnCl_2_.2H_2_O Catalyst at the Reaction Temperature of 60 °C, Molar Ratio of CSO and methanol of 1:30, and Reaction Time of 120 min.

**Figure 3:**
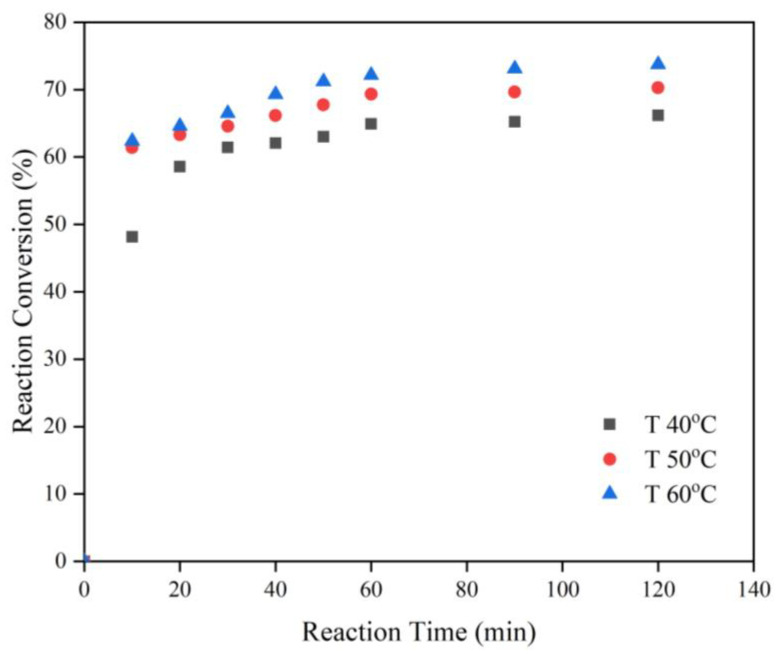
Effect of the Temperature and Reaction Time on the Reaction Conversion of FFA Esterification in CSO over SnCl_2_.2H_2_O Catalyst at the Molar Ratio of CSO: methanol of 1:30 and Catalyst Concentration of 5%.

Based on the stoichiometry, one mole FFA requires one mole methanol to precede the esterification reaction [41]. However, the Fischer esterification reaction is an equilibrium limited reaction. Thus, a great excess of methanol reactant should be introduced to shift the equilibrium towards the product formation [42]. In this work, a fixed CSO to methanol ratio of 1:30 was applied for all the experiments. To intensify the mixing between the reactants and catalyst, the agitation speed was kept at 1000 rpm. The rapid agitation is beneficial to reduce the film thickness between the reactants and promote the mass transfer [42]. The experimental results are demonstrated in Figure 2 and Figure 3.

Figure 2 presents the effect of the catalyst molar ratio on the reaction conversion for the reaction conducted at a fixed reaction temperature, molar ratio of CSO and methanol, and reaction time of 60 °C, 1:30, and 120 min, respectively. The effect of the catalyst concentration was studied at the range of 1–7% *w/w* CSO. Catalyst offers an altered reaction route with lower activation energy. Hence, it causes a higher percentage of collisions between the reactants’ molecules when they reach the minimum energy to react. It can be observed that the reaction conversion was enhanced to 73.75% with an increase in catalyst concentration from 1% to 5%. The higher reaction conversion was accomplished on account of the increased number of active sites available for the reaction [43,44]. Thus, it accelerated the reaction to reach the equilibrium. However, it was revealed that the employment of 7% catalyst did not further raise the reaction conversion. Instead, the conversion tended to slightly decline to 65.85%. This means that the excessive addition of catalyst will not provide a comparative influence on the conversion improvement when the contact process has already arrived at the maximum [45].

Figure 3 exhibits the effects of the temperature and the reaction time on the reaction conversion for the reaction carried out at a fixed catalyst concentration of 5% and molar ratio of CSO: methanol of 1:30. The reaction temperature was examined at 40, 50 and 60 °C and the reaction time was inspected at 0–120 min. It was disclosed that the rising of the temperature brought about the extensively higher reaction conversion. Esterification is an endothermic reaction; therefore the reaction rate increased with the temperature [46]. A rise in the temperature will also improve the translation and the rotation of the reactants’ molecules and lower the liquid viscosity, which will enhance the diffusion rate of the reactants to the active sites of the catalyst [45]. The effective mass transfer has a beneficial impact on the higher total reaction rate and higher reaction conversion. The highest conversion of 73.75% was obtained at 60 °C, which was near to the boiling point of the methanol. A further increase in the temperature at a similar atmospheric pressure will not promote the conversion since it will exceed the boiling point, and hence part of the methanol in the liquid phase will change to the gas phase. The result was in a good agreement with Handayani et al. [47]. The longer the reaction time, the higher the conversion that was attained. However, a sharp acceleration was shown in the first 10 min of the reaction. It was attributed to the high concentration of the reactant at the beginning of the reaction. To determine the optimum process condition which led to the best reaction conversion, analysis using Box–Behnken Design (BBD) in Response Surface Methodology (RSM) was also carried out.

### 3.2. Model Fitting in Box–Behnken Design (BBD)

Response Surface Methodology (RSM) using Box–Behnken Design (BBD) is broadly applied to determine the optimum condition of the variables which results in the desired response. It is also practical for evaluating the effects of the independent variables and the interaction between the independent variables [48]. In this work, BBD was employed to examine the effects and interactions of the independent variables (reaction time, reaction temperature, and catalyst concentration) to determine the optimum condition which produced the highest ester yield and the lowest FFA content in the esterification of CSO using methanol over SnCl_2_.2H_2_O catalyst.

The Box–Behnken response surface design and corresponding response values in this work, including the comparison between the experimental data and the prediction value as well as the errors, are revealed in Table 3. Error is the disparity between the observed and the predictive values, and, accordingly, it can be used to evaluate the accuracy of the model. The error values in this study were calculated in term of mean absolute percentage error (MAPE) as conveyed in Equation (3). It was revealed that the MAPE of the FFA conversion and the FFA content responses were 2.2704% and 3.3410%. The values of MAPE were far less than 10%, indicating the high correctness of the prediction. Generally, values of MAPE below 10% designate a high accuracy of prediction, whereas the values of 10–20%, 20–50%, and higher than 50% imply good, fair, and inaccurate forecasting, respectively [49].

There are various models that are available for the optimization using RSM. In this work, four polynomial models (viz., linear, 2FI or two-factor interaction, quadratic, and cubic) were assessed to decide the most appropriate model to suit the experimental data. The above mentioned models have been extensively studied in the field of bioresources processing research [25,50]. The evaluation of the models was carried out using two different statistical testing methods, i.e., the sequential model (sum of squares) and the model summary tests. Based on the sequential model sum of squares test (Table 4) and the model summary test (Table 5), it was found that the suggested model to optimize the FFA conversion and the FFA content in the case of CSO esterification over SnCl_2_.2H_2_O catalyst was the quadratic model. The quadratic model was designated due to the facts that it provided the lowest p value as indicated in Table 4, and, at the same time, it shown the highest adjusted R^2^ and predicted R^2^ as demonstrated in Table 5.

The empirical correlation of the variables and the response based on the quadratic model resulting from the BBD can be stated in the form of a second order polynomial equation. The general equation for the second order polynomial regression model is written in Equation (4).

(4) $$Y=\beta o+\sum_{i=1}^{k}\left(\beta \mathrm{iXi}\right)+\sum_{i=1}^{k}\left({\beta \mathrm{iiXi}}^{2}\right)+\sum_{ii=1}^{k}\;\sum_{j>1}^{k}\left(\beta \mathrm{ijXiXj}\right)$$

Y indicates the predicted response, βo is a constant, βi is a coefficient for the linear, βii is the coefficient for the quadratic, and βij is the interactive coefficient [29,51]. Thus, the definitive equations for the FFA conversion and FFA content are revealed in Equations (5) and (6), respectively.

(5) $$\begin{array}{ll} FFA\;Conversion\;\left(\%\right)= & 3.47466\;-\;1.29512\;A-0.457250\;B+37.23375\;C\;+\;\\ & 0.000263\;AB+0.106725\;AC-0.005271\;BC\;+\;\\ & 0.011331\;A^{2}+0.002753\;B^{2}-3.76878\;C^{2} \end{array}$$

(6) $$\begin{array}{ll} FFA\;Content\;\left(\%\right)= & 17.746-0.238292\;A-0.4084117\;B+6.85158\;C+\;\\ & 0.000048\;AB+0.19650\;AC-0.000975\;BC+0.002084\;A^{2}+\;\\ & 0.000507\;B^{2}-0.693521\;C^{2} \end{array}$$

where *A*, *B,* and *C* are the temperature (°C), reaction time (min), and catalyst concentration (%), respectively.

### 3.3. Statistical Analysis Using ANOVA

The quadratic model as the most appropriate model was thenceforth analyzed using analysis of variance (ANOVA). The significance of the actual data to the different models based on their associated p-values is displayed in Table 6 and Table 7. Table 6 shows the statistical analysis using ANOVA to predict the FFA conversion in the esterification of CSO. The significance of each constant and the intensity of interaction were proved by the p-value. Influences lower than 0.05 are significant [50]. It can be observed that the F value was 24.37 at the *p*-value < 0.05, denoting that the model was significant. In this investigation, it was discovered that the affecting variables were two linear coefficients (A and C) and one quadratic coefficient (C^2^). This implies that the temperature (A) and catalyst concentration (C) were significant to the model, but the reaction time (B) was insignificant. The adeq precission value is the measurement of the ratio of the signal against the interference, in which the expected ratio is >4. Table 6 demonstrates that the adeq precission was 14.6107, revealing that the model was significant [52]. The lack of fit was 14.08 at a *p*-value of 0.78, which was determined to be significant. It can be suggested that the model is suitable for the prediction of the FFA conversion.

The use of the ANOVA regression model to predict the left over FFA content after the esterification reaction of CSO can be observed in Table 7. The experimental data were analyzed using ANOVA, and the significant regression coefficient was determined based on the *p*-value, in which a *p*-value < 0.05 denotes that the model is significant. The value of adeq precision is the magnitude of the ratio of the signal to the disturbance, wherein the desirable value is >4 [52,53]. This model showed the adeq precision of 14.6107, indicating that the model is accurate.

### 3.4. Optimization of the Process Variables Using BBD

The optimization of the process variables to obtain the targeted response variables was performed using a quadratic model of BBD. Primarily, the influences of the process variables, such as temperature, reaction time, and catalyst concentration, to the response variables, viz., the reaction conversion and the FFA content in the CSO esterification over SnCl_2_.2H_2_O catalyst, were investigated using BBD in RSM. Based on the model selected, analysis of the main effect and the interaction of the process variables to the response variable using 3D RSM was carried out. The resulting 3D graphs were developed from maintaining one constant variable (derived from the midpoint) and varying two other variables. Therefore, the effect of each process variable on the response variable can be identified.

**Figure 4:**
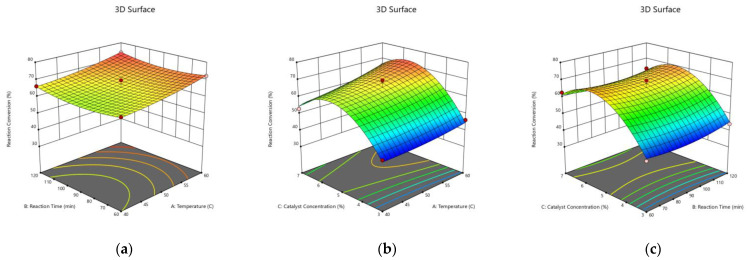
Three Dimensional (3D) Response Surface of the Effects of the Process Variables on the Reaction Conversion. (**a**) Catalyst Concentration of 5%; (**b**) Reaction Time of 90 min; (**c**) Reaction Temperature of 50 °C.

**Figure 5:**
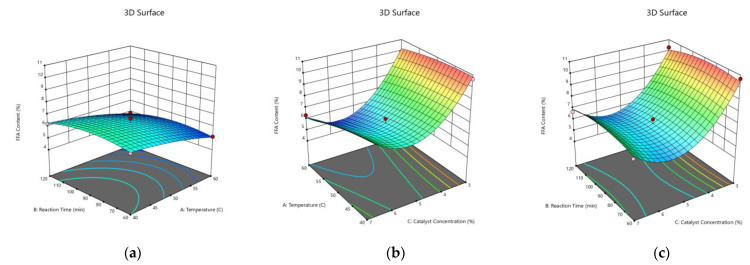
Three Dimensional (3D) Response Surface of the Effects of Process Variables on the FFA Content in after the Undergoing the Esterification Reaction. (**a**) Catalyst Concentration of 5%; (**b**) Reaction Time of 90 min; (**c**) Reaction Temperature of 50 °C.

Figure 4 and Figure 5 disclose that the reaction conversion increased and the FFA content decreased with the temperature up to 60 °C, respectively. The intensification of the catalyst concentration from 3% to 5% enhanced the reaction conversion and diminished the FFA content considerably. This was due to the increased number of reactant molecules which were activated by the carbonyl polarization due to the higher amount of Sn^+2^ catalyst. Hence, the nucleophilic attack by methanol could occur more frequently and effectively, leading to the higher reaction conversion. Oppositely, the leftover FFA content was reduced [54]. There are various proposed mechanisms concerning the carbonyl group activation by tin catalyst, yet the carbonyl polarization will be auspicious when attacked by the hydroxyl group [55]. However, the further increase of the catalyst from 5% to 7% did not provide a meaningful effect in terms of improving the reaction conversion and lessening the FFA content. As a matter of fact, it can be observed that the employment of 7% catalyst increased the FFA content. Marso et al. [56] described how an excessive utilization of the catalyst beyond the optimum concentration could form an emulsion which increased the viscosity and thus hindered the contact between the CSO and the methanol. Consequently, it lowered the reaction conversion. Hence, the residual FFA in the oil was higher.

**Figure 6:**
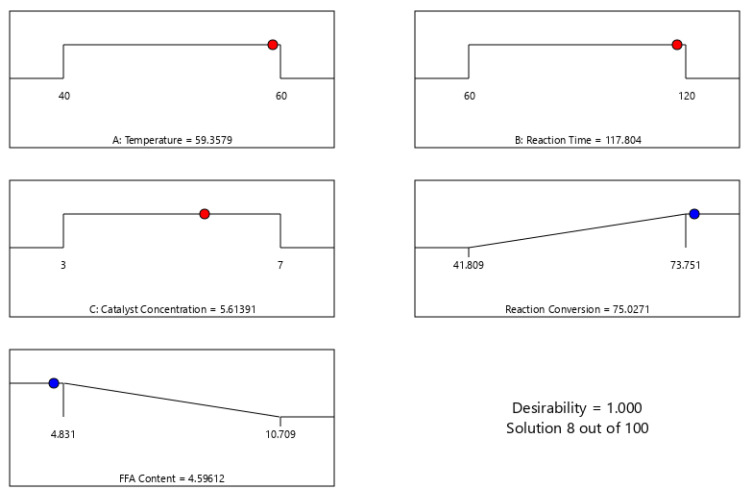
Optimization of Reaction Conversion and FFA Content using BBD Quadratic Model in RSM.

In this study, the Derringer method was utilized to optimize the reaction conversion and the reduction of FFA content via CSO esterification over SnCl_2_.2H_2_O catalyst. The Derringer method is a popular desirability function-based approach to solving a problem comprising a simultaneous optimization of several response variables. Derringer and Suich [57] modified the previous Harrington’s procedure by converting the response into a desirability function [58]. The values of desirability functions are between 0 and 1. Mathematically, the general approach is to convert each response into an individual desirability function (d) that varies over the range 0 ≤ d ≤ 1 [59]. The value of 0 implies that the factors present unfavorable response. On the other hand, the value of 1 relates to the optimal condition of the examined factors and indicates that the responses are at their targets. This approach simplifies the multivariate optimization. Due to its simplicity and flexibility, the Derringer desirability function has been broadly applied in multiple responses optimization to find out the independent variables condition which brings about the optimal values of the response variables [60]. Based on the optimization process, Figure 6 reveals that the optimum reaction conversion and FFA content were 75.03% and 4.59%, respectively, which were achieved at the following operation condition: a reaction temperature of 59.36 °C, a reaction time of 117.8 min, and a catalyst concentration of 5.61%. The value of desirability obtained was 1, indicating the optimal condition of the studied parameters. This result was slightly lower than that for the similar reaction which was conducted using sulfuric acid catalyst at the reaction temperature, catalyst loading, and reaction time of 59.09 °C, 1.98% *g/g* CSO, and 119.95 min, respectively, resulting in the reaction conversion of 78.27% and the FFA content of 4% [25]. Despite this slight lower conversion, the application of heterogeneous SnCl_2_.2H_2_O catalyst is greatly preferable to the sulfuric acid catalyst since it is more environmentally friendly, reusable, less corrosive, and easier in handling and separation. The result of this work offers a green alternative of synthesizing renewable bio based fatty ester from CSO as precursor of epoxy ester plasticizer.

## 4. Conclusions

The esterification of FFA in *Calophyllum inophyllum* Seed Oil (CSO) using methanol in the presence of SnCl_2_.2H_2_O catalyst has been conducted as an alternative way to produce fatty acid ester as a green precursor of epoxy ester plasticizer. In this investigation, the interactive and individual effects from three experimental variables (temperature, reaction time, and catalyst concentration) on reaction conversion and residual free fatty acid (FFA) content were studied by employing the Box–Behnken Design (BBD) of Response Surface Methodology (RSM) technique. The quadratic model in BBD was selected for the optimization of the reaction conversion and the decreasing of the FFA content. The BBD analysis showed that the optimum FFA conversion and residual FFA content were 75.03% and 4.59%, respectively, attained at the following process condition: a reaction temperature of 59.36 °C, a reaction time of 117.80 min, and a catalyst concentration of 5.61%. The fatty acid ester generated is subsequently ready for the further epoxidation process to produce epoxy plasticizer in polymeric material production.

## Figures and Tables

**Table 1:** Independent Variables Range and Level Used in BBD Experimental Design.

Independent Variable	Factor	Coded Level		
		−1	0	1
Temperature (°C)	A	40	50	60
Reaction Time (min)	B	60	90	120
Catalyst Concentration (%)	C	3	5	7

**Table 2:** Design of the Randomized Response Model.

Run	Factor A Temperature (°C)	Factor B Reaction Time (min)	Factor C Catalyst Concentration (%)
1	40	120	5
2	40	60	5
3	60	90	3
4	40	90	7
5	60	90	7
6	50	120	3
7	60	120	5
8	50	60	7
9	50	90	5
10	40	90	3
11	60	60	5
12	50	60	3
13	50	90	5
14	50	120	7
15	50	90	5

**Table 3:** The Box–Behnken Response Surface Design and Corresponding Response Values.

Run	Temperature (°C) A	Reaction Time (min) B	Catalyst Concentration (%) C	FFA Conversion %		Error (MAPE) %	FFA Content (%)		Error (MAPE) %
				Experiment	Prediction		Experiment	Prediction	
1	40	120	5	66.161	65.963	0.2987	6.227	6.264	0.5862
2	40	60	5	64.896	64.619	0.4267	6.460	6.511	0.7895
3	60	90	3	46.237	44.695	3.3348	9.894	10.178	2.8704
4	40	90	7	52.878	54.420	2.9160	8.672	8.388	3.2749
5	60	90	7	65.528	66.595	1.6289	6.344	6.148	3.0974
6	50	120	3	44.023	45.288	2.8735	10.301	10.068	2.2619
7	60	120	5	73.751	74.028	0.3755	4.831	4.780	1.0557
8	50	60	7	62.682	61.417	2.0181	6.867	7.100	3.3930
9	50	90	5	63.631	65.634	2.0181	6.693	6.324	5.5132
10	40	90	3	42.125	41.058	2.5339	10.650	10.847	1.8451
11	60	60	5	72.170	72.368	0.2738	5.122	5.086	0.7126
12	50	60	3	41.809	43.153	3.2153	10.709	10.462	2.3111
13	50	90	5	69.640	65.634	5.7524	5.587	6.324	13.1967
14	50	120	7	63.631	62.287	2.1125	6.693	6.941	3.6979
15	50	90	5	63.631	65.634	3.1478	6.693	6.324	5.5087
	MAPE (%)					2.2704			3.3410

**Table 4:** Sequential Model (Sum of Squares) Test.

Component	Sum of Square	Degree of Freedom	Mean Square	F-Value	*p*-Value	Remarks
Sequential (Sum of Square) for the FFA Conversion						
Mean	53,138.62	1	53,138.62			
Linear	751.26	3	250.42	2.87	0.09	
2FI	18.65	3	6.22	0.05	0.98	
Quadratic	903.67	3	301.22	39.48	0.0007	Suggested
Cubic	14.08	3	4.69	0.39	0.7758	Aliased
Residual	24.07	2	12.04			
Total	54,850.36	15	3656.69			
Sequential (Sum of Square) for the FFA Content						
Mean	832.43	1	832.43			
Linear	25.44	3	8.48	2.87	0.09	
2FI	0.63	3	0.21	0.05	0.98	
Quadratic	30.60	3	10.20	39.44	0.0007	Suggested
Cubic	0.48	3	0.16	0.39	0.7756	Aliased
Residual	0.82	2	0.41			
Total	890.40	15	59.36			

**Table 5:** Model Summary Test.

Component	Standard Deviation	R^2^	Adjusted R^2^	Predicted R^2^	Press	Remarks
Model Summary for the FFA Conversion						
Linear	9.34	0.44	0.29	−0.12	1921.57	
2FI	10.85	0.45	0.04	−1.59	4446.52	
Quadratic	2.76	0.98	0.94	0.84	279.43	Suggested
Cubic	3.47	0.99	0.90		*	Aliased
Model Summary for the FFA Content						
Linear	1.72	0.44	0.29	−0.12	65.07	
2FI	2.00	0.45	0.04	−1.59	150.57	
Quadratic	0.51	0.98	0.94	0.84	9.47	Suggested
Cubic	0.64	0.99	0.90		*	Aliased

* means not defined.

**Table 6:** Analysis of the Variance and Regression Coefficients of the BBD Quadratic Model to Predict the FFA Conversion.

Source	Sum of Square	DF	Mean Square	F Value	*p*-Value	
Model	1673.58	9	185.95	24.37	0.00	Significant
A Temperature (°C)	125.03	1	125.03	16.39	0.01	
B Reaction Time (min)	4.51	1	4.51	0.59	0.48	
C Catalyst Concentration (%)	621.72	1	621.72	81.48	0.00	
AB	0.03	1	0.03	0.003	0.96	
AC	18.22	1	18.22	2.39	0.18	
BC	0.40	1	0.40	0.05	0.83	
A^2^	4.74	1	4.74	0.62	0.47	
B^2^	22.66	1	22.66	2.97	0.15	
C^2^	839.11	1	839.11	109.97	0.00	
*Residual*	38.15	5	7.63			
*Lack of Fit*	14.08	3	14.08	0.39	0.78	Not Significant
*Pure Error*	24.08	2	12.04			
*Cor Total*	1711.73	14				
*Adeq Precision*	14.62					
R^2^	0.98					

**Table 7:** Analysis of the Variance and Regression Coefficients of the BBD Quadratic Model to Predict the FFA Content.

Source	Sum of Square	DF	Mean Square	F Value	*p*-Value	
Model	56.67	9	6.30	24.35	0.00	Significant
X_1_	4.23	1	4.23	16.36	0.01	
X_2_	0.15	1	0.15	0.59	0.48	
X_3_	21.05	1	21.05	81.41	0.00	
X_12_	0.00	1	0.00	0.00	0.96	
X_13_	0.62	1	0.62	2.39	0.18	
X_23_	0.01	1	0.01	0.05	0.83	
X_1_^2^	0.16	1	0.16	0.62	0.47	
X_2_^2^	0.77	1	0.77	2.97	0.15	
X_3_^2^	28.41	1	28.41	109.88	0.00	
*Residual*	1.29	5	0.26			
*Lack of Fit*	0.48	3	0.16	0.39	0.78	Not Significant
*Pure Error*	0.82	2	0.41			
Cor Total	57.96	14				
R^2^	0.98					
*Adeq Precision*	14.61					

## Data Availability

Not applicable.
